# Nutrition-specific and sensitive drivers of poor child nutrition in Kilte Awlaelo-Health and Demographic Surveillance Site, Tigray, Northern Ethiopia: implications for public health nutrition in resource-poor settings

**DOI:** 10.1080/16549716.2018.1556572

**Published:** 2019-01-14

**Authors:** Semaw Ferede Abera, Eva Johanna Kantelhardt, Afewrok Mulugeta Bezabih, Alemseged Aregay Gebru, Gebisa Ejeta, Judith Lauvai, Andreas Wienke, Veronika Scherbaum

**Affiliations:** a Institute of Biological Chemistry and Nutrition, University of Hohenheim, Stuttgart, Germany; b Food Security Center, University of Hohenheim, Stuttgart, Germany; c School of Public Health, College of Health Sciences, Mekelle University, Mekelle, Ethiopia; d Kilte Awlaelo- Health and Demographic Surveillance Site, College of Health Sciences, Mekelle University, Mekelle, Ethiopia; e Institute of Medical Epidemiology, Biostatistics, and Informatics, Faculty of Medicine, Martin-Luther-University, Halle, Germany; f Department of Gynaecology, Faculty of Medicine, Martin-Luther-University, Halle, Germany; g Department of Agronomy, Purdue University, West Lafayette, IN, USA

**Keywords:** Child malnutrition, nutrition-specific drivers, nutrition-sensitive drivers, KA-HDSS, Ethiopia

## Abstract

**Background**: Child undernutrition is a prevalent health problem and poses various short and long-term consequences.

**Objective**: This study seeks to investigate the burden of child undernutrition and its drivers in Kilte Awlaelo-Health and Demographic Surveillance Site, Tigray, northern Ethiopia.

**Methods**: In 2015, cross-sectional data were collected from 1,525 children aged 6–23 months. Maternal and child nutritional status was assessed using the mid upper arm circumference. Child’s dietary diversity score was calculated using 24-hours dietary recall method. Log-binomial regression and partial proportional odds model were fitted to examine the drivers of poor child nutrition and child dietary diversity (CDD), respectively.

**Results**: The burden of undernutrition and inadequate CDD was 13.7% (95% CI: 12.1–15.5%) and 81.3% (95%CI: 79.2–83.1%), respectively. Maternal undernutrition (adjusted prevalence ratio, adjPR = 1.47; 95%CI: 1.14–1.89), low CDD (adjPR = 1.90; 95%CI: 1.22–2.97), and morbidity (adjPR = 1.83; 95%CI: 1.15–2.92) were the nutrition-specific drivers of child undernutrition. The nutrition-sensitive drivers were poverty (compared to the poorest, adjPR poor = 0.65 [95%CI:0.45–0.93], adjPR medium = 0.64 [95%CI: 0.44–0.93], adjPR wealthy = 0.46 [95%CI: 0.30–0.70], and adjPR wealthiest = 0.53 [95%CI: 0.34–0.82]), larger family size (adjPR = 1.10; 95%CI: 1.02–1.18), household head’s employment insecurity (adjPR = 2.10; 95%CI: 1.43–3.09), and residing in highlands (adjPR = 1.93; 95%CI: 1.36–2.75). The data show that higher CDD was positively associated with wealth (OR wealthy = 3.06 [95%CI: 1.88–4.99], OR wealthiest = 2.57 [95%CI: 1.53–4.31]), but it was inversely associated with lack of diverse food crops production in highlands (OR = 0.23; 95%CI: 0.10–0.57]).

**Conclusions**: Our findings suggest that the burden of poor child nutrition is very high in the study area. Multi-sectoral collaboration and cross-disciplinary interventions between agriculture, nutrition and health sectors are recommended to address child undernutrition in resource poor and food insecure rural communities of similar settings.

## Background

Child undernutrition is the leading cause of child morbidity and mortality globally [1,2]. It is still a persistently prevalent health problem in Ethiopia [3–8] even though its burden shows a declining trend [9]. According to the Ethiopian Demographic and Health Survey (EDHS) 2000 and 2016 child wasting, an indicator of acute food deficit, showed only a 2% decline at national level, and persisted at 11.1% in Tigray regional state [9,10].

Multiple factors and their complex interactions result in child undernutrition. In brief, the literature shows that high household wealth status, maternal access to health services, child dietary diversification, food security, access to improved water and sanitation, better housing quality, prevention and timely treatment of acute malnutrition are strongly associated with optimal child nutrition and survival [11–22]. On the other hand, maternal nutritional insult, larger family size with short birth interval, the absence of diverse agricultural food crop production, living in highlands and childhood morbidity, were reported to be associated with child undernutrition [11,14,15,22–30].

Nutritional wellbeing and survival of children could also be jeopardized through household shocks after illnesses or mortality of adult household members. Children living in households with a substantial loss of household earnings, due to a high burden of sickness-induced loss of employment, were found to be severely undernourished [30]. A significantly higher risk of falling into poverty and food insecurity was also observed for households which experienced adult mortality from both chronic diseases as well as acute infectious diseases [31–33] and this could also negatively impact child nutritional status and wellbeing.

Left unaddressed, child undernutrition has several short and long-term consequences to those who are affected and their future generations [11]. Adverse early life due to child undernutrition was related to lower human capital, with a likely intergenerational transmission of poverty (IGTP) [34,35], and higher risks of developing non-communicable diseases (NCDs) during adulthood [34,36,37]. Therefore, ensuring the nutritional wellbeing and survival of children, particularly during the first 1000 days of life, is one of the substantiated public health strategies recommended for reducing present and future disease burden caused by malnutrition [2,38,39]. The main objectives of this study were to assess the burden and nutrition-specific and sensitive drivers of undernutrition as well as dietary diversity among 6–23 months old children in Kilte Awlaelo-Health and Demographic Surveillance Site (KA-HDSS), Tigray, northern Ethiopia.

## Methods

### Setting, study design and data sources

Detailed descriptions of the surveillance site (KA-HDSS) have been published in prior research works [40,41]. This study used cross-sectional data collected during the second census of the site, which was held 5 years after its establishment in 2009. The census data collects several socio-demographic, economic, environmental, and public health-related data. Using the site’s platform and its convenience for implementing other researches, baseline nutritional survey data of mothers and their children were collected as an add-on project. Concisely stated, nutrition-specific drivers refer to the immediate determinants of child nutrition and development, whereas nutrition-sensitive drivers refer to the underlying determinants mentioned in the UNICEF’s framework for child nutrition, health and survival [11,38].

### Study population

All the 1,525 children aged 6–23 months form the study population.

### Measurement of variables

Nutritional status of children and their corresponding mothers was assessed using the mid upper arm circumference (MUAC) measured in centimetre (cm). Maternal MUAC below 23 cm [29,42] is categorized as undernutrition and a maternal MUAC below 21 cm [42] as severe undernutrition. Studies have shown that measuring nutritional status of non-pregnant mothers using MUAC can be a good substitute for body mass index [42–44]. Since MUAC is significantly age and sex dependent, particularly for children < 24 months, the decision of determining nutritional status of children by absolute MUAC estimates is problematic and needs to be adjusted for these factors [45–48]. A study in 2018, based on 255,623 measurements of 19 surveys found that the estimates of acute undernutrition by age-sex adjusted MUAC and weight for-height/length Z-score (WHZ) was similar, unlike the estimates from the absolute MUAC values which were discrepantly lower [49]. Acknowledging the relevance of the methodological recommendations of the cited studies, in this study, children’s MUAC values were transformed to standardized z-score based MUAC (MUACZ) using WHO Anthro 2011 adjusting the age and sex of each child [50]. Then, child undernutrition was defined if the MUAC was <-2 z-score (MUAC <-3 z-score being defined as severe undernutrition and moderate undernutrition if the MUAC is <-2 to ≥-3 z-score). Biologically implausible MUACZ score values were dropped if the values fell out of the range of −5 and +5 [50]. Accordingly, two observations were dropped because their corresponding MUACZ scores were < −5.

Child’s dietary diversity score (CDDS) was calculated out of seven food groups using the 24-hrs dietary recall method [51]. The responses to each of the seven food groups were dichotomized (‘1’ if a given food group was consumed or ‘0’ if it was not consumed) and summed up to obtain the child dietary diversity score with values ranging from a minimum of 0 to a maximum of 7. This procedure was done using the World Health Organization (WHO) technique (51). Consumption of each food group was also disaggregated by age group of the children according to the WHO recommendation [52]. Then, in the model that examined the drivers of child undernutrition, child dietary diversity score was recoded as adequate (consumption of ≥4 food groups/day) and inadequate (consumption of <4 food groups/day) because consumption of four or more food groups was related to better diet quality [51,52]. However, in a separate model which analyzed the drivers of dietary intake of the young children, CDDS was categorized as low (consumption of <4 food groups), medium (consumption of 4 to 5 food groups), and high (consumption of ≥6 food groups) [53–55].

Socio-economic position was measured using wealth index, applying principal component analysis [56,57], from a wide range of variables such as accessibility to improved water and sanitation services as defined by the WHO/UNICEF Joint Monitoring Program (JMP) ladders [58], levels of housing quality computed by replicating a prior research work [59], availability and quantity of agricultural ownership (farmland, bee hive, cart, livestock and food crops produced), access to electricity, media (created from single or joint ownership of TV or radio or phone), and other household ownerships like a bed with sofa, use of non-biomass energy source for food cooking etc. Detail of our methodology for generating wealth index is provided in Appendix D of the supplementary material.

The variable maternal health-seeking practice (mHSP) was constructed from two variables (current use of modern contraceptive and maternal tetanus toxoid immunization during pregnancy) and its values range from 0 to 2. This proxy variable was then dichotomized as ‘good practice’ if the score was 2 (mother used both maternal health services) and ‘poor practice’ if the score was less than 2 (mother used either none or only one of the two maternal health services). Altitudinal location of households was measured using geographic positioning system (GPS) and classified as highland (≥2, 300 meter) and low/midland (<2, 300 meter) [41]. Causes of adult death (CoD) were identified using physician review method and classified according to International Classification of Diseases version 10 (ICD-10) [60]. In this paper, the specific causes of death are then operationally reclassified as chronic causes (NCDs including cancer, cardio-vascular diseases, diabetes, and chronic lung diseases as well as chronic infectious diseases including TB and HIV/AIDS) and other causes (all acute infectious diseases, external causes, unspecified causes, undetermined and pregnancy-related CoD) primarily based on the chronicity of the CoD. The rationale underlying this classification is due to the difference in the length of the duration of illness of the CoD, which is primarily determined by the nature of the expected course of duration from clinical-stage development to the time of death. The assumption was that mortality from chronic CoD might have caused a differentially immense household level negative impacts, over the possibly longer period of illness of the deceased adults, and thereby distinctively expose the young children to undernutrition [30,61,62].

### Statistical analyses

Modeling of child undernutrition was performed using generalized linear regression with a log link and binomial distribution. This statistical procedure was chosen, compared to the most commonly used binary logistic regression, because if the outcome is not rare (outcome > 10%), odds ratio (OR) can no longer approximate the risk ratio and is not an appropriate measure of association. Therefore, in studies with common outcomes, prevalence ratio (PR) should be used to measure an association [63,64]. In this study, the prevalence of child undernutrition (defined by MUAC <-2 z-score) was 13.7% which indicates that, in the study setting, the outcome of our interest is not a rare condition. Next, a proportional odds model (POM) was performed to identify the factors associated with child dietary diversity score. This statistical procedure was chosen as opposed to other modeling options, such as multinomial model or multivariable binary logistic regression model, because CDDS is a polychotomous variable with meaningfully inherent order. Thus, multinomial procedure was not used. POM is a more parsimonious, efficient and appropriate model than running multiple separate binary logistic regression models [65,66]. The POM convergence problem was avoided using the ‘*difficult*’ command option in Stata 13.0 [64]. Then, *brant test* was used to assess the proportional odds/parallel-lines assumption of each variable. Using this test, the variable ‘geographic location’ had a significant p-value indicating that this variable violated the parallel-lines assumption of POM and hence a partial proportional odds model (PPOM), with gamma parametrization method, was fitted by un-constraining this variable and constraining all other variables. The interpretation of ‘geographic location’ variable based on the PPOM model has enabled us to identify its pattern of association with the outcome variable, which otherwise would have remained obscured in the POM. Evidence of multicollinearity was assessed using variance inflation factor (VIF) at cut-off value of greater than 10 [67] and no collinearity was found as reported in Appendix F1 of the supplementary material. In the univariable analysis of both models, all variables with a p value of < 0.25 were selected [68,69] and fitted into the multivariable models in which statistical significance was declared at p value of <0.05.

## Results

Details of household level including socio-economic, agricultural, and epi-demographic characteristics are presented in Table A1. Generally, increasing pattern of quintile wealth index was observed for the households with access to infrastructural service and improved public health indicators such as availability of electricity, improved housing quality, kitchen and non-biomass cooking fuel (Table A1).

### Causes of adult death

Of the total 1,525 surveyed households, 91 (6 %) of the households had a history of death of an adult household member who deceased between 2009 and 2015. About half of the adult deaths, 45 (49.5%) were due to chronic diseases and the remaining 46 were due to other causes.

### Young child undernutrition

This study focused on children from 6 months to 23 months of age. Almost half of the children were female (50.5 %). The mean (and standard deviation, SD) age of the study participants was 13.5 ± 4.9 months (13.5 ± 5.0 months for females and 13.4 ± 4.8 months for males). Out of the total 1, 525 study participants, 209 children, 13.7% (95% CI: 12.1–15.5%), were undernourished of which 43 (20.6%) were of a severe form. The burden of undernutrition was higher among male children compared to females (15.9% vs 11.6%, p = 0.014). The mean (SD) child MUACZ score was −0.87 (1.13). The distribution of MUACZ score of the study children was left shifted as compared with the Z-scores of WHO standard (Figure B1).

Based on MUAC cut off value of < 23 cm 39.9% (95% CI: 37.5–42.4) of the children’s mothers were undernourished and 102 (16.9%) of these mothers were severely undernourished (MUAC < 21 cm). Access to adequate child dietary diversity was 18.7% (95%CI: 16.9–20.8%). Consumption of fruits and vegetables rich in vitamin A (3.3 % vs 10.1 %; p = 0.002) and dairy food (19.4% vs 31.5%, p < 0.001) was significantly lower for the undernourished children compared to those who were not (data not shown). As presented in Table 1, the burden of undernutrition was higher among young children who had lower mean dietary diversity intake and whose mothers were undernourished. Child undernutrition was also relatively high among those who were living in households which had lower wealth quintiles, higher mean family size, located in highlands, and living in households which experienced symptomatic morbidity (cough, diarrhea, fever, and/or any other symptom) and produced no diverse food crops (Table 1).

**Table 1.** Nutritional status of children by selected independent variables, KA-HDSS, Tigray, northern Ethiopia (n = 1, 525).

Characteristics	Categories	Row distribution of Nutritional status of children, n (%)		Column total, n (%)
		MUAC ≥-2 *z*-score	MUAC <-2 *z*-score	
Residence	Semi-urban	80 (94.1)	5 (5.9)	85 (5.6)
	Rural	1,236 (85.8)	204 (14.2)	1, 440 (94.4)
Sex of household head	Male	1,137 (85.8)	188 (14.2)	1, 325 (86.9)
	Female	179 (89.5)	21 (10.5)	200 (13.1)
Sex of child	Male	635 (84.1)	120 (15.9)	755 (49.5)
	Female	681 (88.4)	89 (11.6)	770 (50.5)
Educational status of household head	No formal education	886 (85.8)	146 (14.2)	1, 032 (67.7)
	Formal education	428 (87.2)	63 (12.8)	491 (3.2)
	Missing			2 (0.1)
Occupation of household head	Farmer/housewife	1,123 (86.9)	169 (13.1)	1,292 (84.7)
	Daily laborer	110 (76.9)	33 (23.1)	143 (9.4)
	Government employee and others	83 (92.2)	7 (7.8)	90 (5.9)
Mean age of household head in years (SD)		42.3 (11.1)	44.8 (11.5)	1, 525
Mean child dietary diversity score		2.2 (1.5)	1.9 (1.3)	1, 525
Maternal MUAC	normal	814 (89.6)	94 (10.4)	908 (60.1)
	Undernourished	491 (81.3)	113 (18.7)	604 (39.9)
History of adult death	No history adult death	1,234 (86.0)	200 (14.0)	1, 434 (94.0)
	Yes-from chronic diseases	40 (88.9)	5 (11.1)	45 (2.9)
	Yes- from acute infectious diseases, injuries and other causes	42 (91.3)	4 (8.7)	46 (3.0)
Mean quintile wealth status (SD)		3.1 (1.4)	2.5 (1.4)	1, 483
Geographic location	Low/midland	906 (87.3)	132 (12.7)	1, 038 (68.1)
	Highland	306 (81.6)	69 (18.4)	375 (24.6)
	Missing			112 (7.3)
Morbidity history in the past 2 weeks	No	1,274 (86.7)	195 (13.3)	1, 469 (96.3)
	Yes	41 (74.5)	14 (25.5)	55 (3.6)
	Missing			1 (0.07)
Mean household size (SD)		6.2 (2.0)	6.8 (1.9)	1, 525
No crop/monocrop production	Yes	575 (83.8)	111 (16.2)	686 (45.0)
	No	741 (88.3)	98 (11.7)	839 (55.0)

### Patterns of nutritional outcomes and morbidity by age and sex

**Figure 1.**
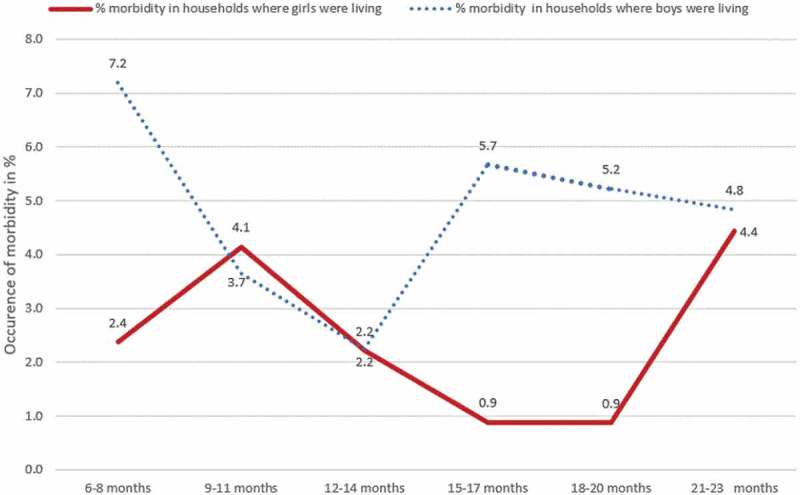
Distribution of households’ experience of morbidity by sex and age of children, KA-HDSS, Tigray, northern Ethiopia (n = 1, 525).

The percentage of occurrence of morbidity was higher in the households where the boys resided as compared to the households where the girls resided, 4.8 % vs 2.5 % respectively. Boys were living in 65.5 % of the total households which reported occurrence of morbidity. Except for the boys in the age groups of 9–11 months and 12–14 months, reports of occurrence of morbidity were lower for the girls compared to all other age groups (Figure 1).

**Figure 2.**
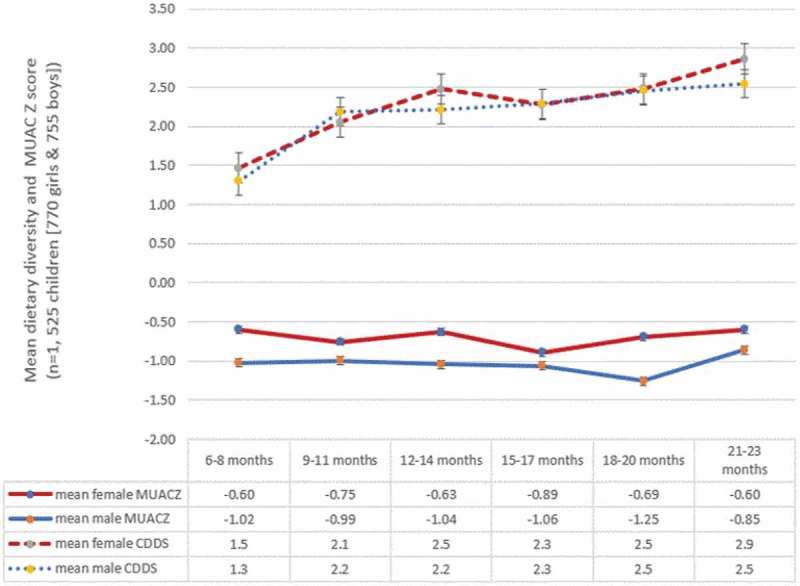
Patterns of mean child dietary diversity score (CDDS) and MUACZ score by sex and age of children, KA-HDSS, Tigray, northern Ethiopia (n = 1, 525).

As shown in Figure 2, the mean MUACZ score values of boys was consistently lower than that of girls across all age groups. In addition, the mean CDDS of girls was either higher or equal to that of the boys except for the 9–11 months age group (Figure 2).

### Determinants associated with child undernutrition

We found that various determinants were significantly associated with child undernutrition including: maternal undernutrition (adjPR = 1.47; 95%CI: 1.14–1.89), inadequate child dietary diversity (adjPR = 1.90; 95%CI: 1.22–2.97), daily laborer occupation of household head (adjPR = 2.10; 95%CI: 1.43–3.09), and occurrence of morbidity among a family member (adjPR = 1.83; 95%CI: 1.15–2.92) (Table 2). Furthermore, higher family size (adjPR = 1.10; 95%CI: 1.02–1.18) and residing in highlands (adjPR = 1.93; 95%CI: 1.36–2.75) were also significantly associated with a higher burden of undernutrition. However, the effect of highland geographic location was significantly modified by the diverse food cropping practice of households. Compared to lowlander children living in households with no crop or monocrop production, highlander children who lived in households that produced more than one type of food crop had a significantly lower prevalence of undernutrition (adjPR = 0.42; 95%CI: 0.23–0.77) (Table 2).

**Table 2.** Prevalence ratios (PR) for the determinants of child undernutrition using crude and adjusted (for all the variables in the table) GLM log-binomial model, KA-HDSS, Tigray, northern Ethiopia (n = 1, 525).

Variables	Categories	Undernutrition			
		Crude PR (95% CI)	p-value	Adjusted PR (95% CI)	p-value
Residence	Urban	1.00		1.00	
	Rural	2.41 (1.02, 4.45)	0.045	1.77 (0.62, 5.04)	0.286
Sex of household head	Male	1.00		1.00	
	Female	0.74 (0.48, 1.13)	0.166	0.86 (0.56, 1.31)	0.472
Maternal health-seeking practice	Poor	1.00		1.00	
	Good	0.76 (0.55, 1.05)	0.098	0.92 (0.66, 1.27)	0.615
Age of household head (5-year increase)		1.08 (1.03, 1.14)	0.002	1.03 (0.97, 1.10)	0.358
Occupation of household head	Farmer	1.00		1.00	
	Daily laborer	1.76 (1.27, 2.46)	0.001	2.10 (1.43, 3.09)	<0.001
	Government employee and other occupations	0.60 (0.29, 1.23)	0.160	1.75 (0.75, 4.08)	0.196
Child dietary diversity score	Adequate	1.00		1.00	
	Inadequate	1.87 (1.23, 2.82)	0.003	1.90 (1.22, 2.97)	0.005
Maternal undernutrition	No	1.00		1.00	
	Yes	1.81 (1.40, 2.33)	<0.001	1.47 (1.14, 1.89)	0.003
Wealth index	Poorest	1.00		1.00	
	Poor	0.54 (0.38, 0.77)	0.001	0.65 (0.45, 0.93)	0.020
	Medium	0.52 (0.36, 0.74)	<0.001	0.64 (0.44, 0.93)	0.019
	Wealthy	0.35 (0.23, 0.54)	<0.001	0.46 (0.30, 0.70)	<0.001
	Wealthiest	0.42 (0.28, 0.62)	<0.001	0.53 (0.34, 0.82)	0.004
History of adult death in household	No history of death	1.00		1.00	
	Chronic diseases	0.80 (0.35, 1.84)	0.594	0.96 (0.42, 2.17)	0.918
	Acute, external and other causes	0.62 (0.24, 1.60)	0.327	0.61 (0.24, 1.53)	0.292
Geographic location	Lowland	1.00		1.00	
	Highland	1.45 (1.11, 1.89)	0.007	1.93 (1.36, 2.75)	<0.001
Two weeks history of morbidity	No	1.00		1.00	
	Yes	1.92 (1.20, 3.07)	0.007	1.83 (1.15, 2.92)	0.012
Household size		1.13 (1.06, 1.20)	<0.001	1.10 (1.02, 1.18)	0.016
No crop or monocrop production	Yes	1.00		1.00	
	No	0.72 (0.56, 0.93)	0.011	1.00 (0.70, 1.43)	0.999
Highland#variety crop production (yes)		0.56 (0.35, 0.90)	0.018	0.42 (0.23, 0.77)	0.005

In comparison with the children who lived in the poorest households, those who lived in the poor, medium, wealthy, and wealthiest households had 0.65, 0.64, 0.46, and 0.53 times lower risk of child undernutrition. Moreover, history of adult death from both chronic and other CoD were not found to be associated with child undernutrition (Table 2).

### Child dietary diversity by sex and age

Majority of the study participants, 81.3% (95% CI: 79.2, 83.1), had an inadequate dietary diversity. High child dietary diversity (≥6 food groups per day) was commonly observed among children whose household heads were married, wealthier households and children residing in low or midland geographic location (Table C1). The least consumed food groups among children in this study were fleshy (6.2 %) and vitamin A rich fruits and vegetables (9.2 %). Whereas, consumption of grains, roots, and tubers (89.8%) and eggs (43%) were considerably higher. Other food groups such as dairy foods, legumes and nuts, and other fruits and vegetables were consumed to 29.8 %, 23.3 % and 14.4 %, respectively. There was no significant difference found among consumption of each food group by sex, except for consumption of eggs, which was found to be more common among the female children (45.9% vs 40%, p = 0.018).

**Figure 3.**
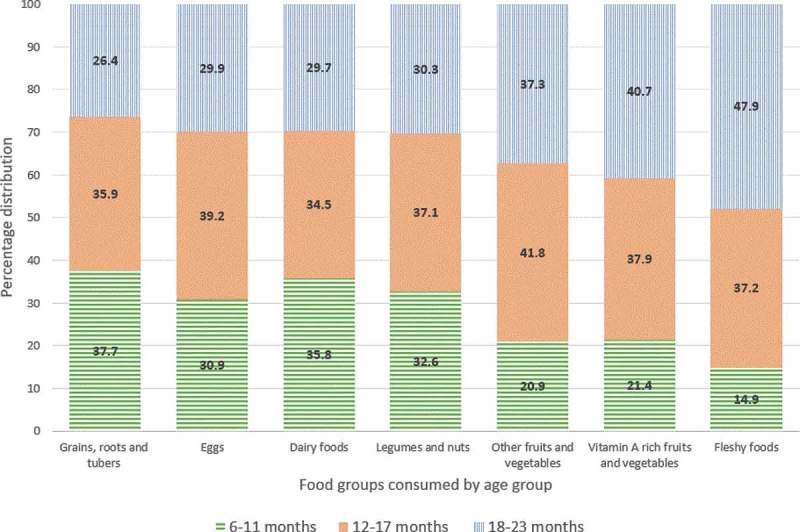
Proportion of children who consumed each food group per day by age group, KA-HDSS, Tigray, northern Ethiopia (n = 1,525).

The consumption of each food group when disaggregated by age group of the children showed statistical significance (p < 0.01). We found that dairy foods, eggs, grains, roots and tubers were more likely to be offered to the younger age groups, while fleshy and vitamin A rich foods were more frequently consumed by the oldest age group (Figure 3).

### Determinants associated with child dietary diversity (CDD)

Children from the wealthiest and wealthy households had 2.57 (OR = 2.57; 95% CI: 1.53–4.31) and 3.06 (OR = 3.06; 95% CI: 1.88–4.99) times greater odds of having a higher dietary diversity compared to children from poorest households, respectively, given the other variables are held constant. Another factor that influenced the likelihood of a lower dietary diversity was when the household head was not in a marital relationship, but this association did not fully rule out the role of chance (Table 3).

**Table 3.** Results from partial proportional odds model (POM) using child dietary diversity score with three ordered categories, KA-HDSS, Tigray, northern Ethiopia (n = 1, 525).

Variables	Categories	High CDDS vs (low and medium CDDS)		High and medium CDDS vs (low CDDS)	
		Adjusted OR (95% CI)	p-value	Adjusted OR (95% CI)	p-value
Household size		1.05 (0.96, 1.13)	0.297	1.05 (0.96, 1.13)	0.297
Maternal health-seeking practice	poor practice	1.00		1.00	
	Good practice	1.07 (0.78, 1.45)	0.678	1.07 (0.78, 1.45)	0.678
Household head occupation	Farmer				
	Daily laborer	0.77 (0.46, 1.30)	0.330	0.77 (0.46, 1.30)	0.330
	Govt. employee and all others	0.88 (0.47, 1.67)	0.704	0.88 (0.47, 1.67)	0.704
Wealth status	Poorest	1.00		1.00	
	Poor	1.09 (0.64, 1.86)	0.759	1.09 (0.64, 1.86)	0.759
	Medium	1.49 (0.90, 2.46)	0.124	1.49 (0.90, 2.46)	0.124
	Wealthy	3.06 (1.88, 4.99)	<0.001	3.06 (1.88, 4.99)	<0.001
	Wealthiest	2.57 (1.53, 4.31)	<0.001	2.57 (1.53, 4.31)	<0.001
History of adult death	No history of adult death	1.00		1.00	
	Death from chronic diseases	1.08 (0.51, 2.27)	0.841	1.08 (0.51, 2.27)	0.841
	Death from acute, external and other causes	1.20 (0.54, 2.65)	0.662	1.20 (0.54, 2.65)	0.662
Geographic location*	Low/midland	1.00		1.00	
	Highland	0.23 (0.10, 0.57)	0.002	1.17 (0.74, 1.84)	0.510
Farmland size	≤2 ha	1.00		1.00	
	>2 ha	1.28 (0.91, 1.80)	0.151	1.28 (0.91, 1.80)	0.151
Variety crop production (yes)#highland geographic location		2.92 (1.75, 4.85)	<0.001	2.92 (1.75, 4.85)	<0.001
Marital status of household head	Married	1.00		1.00	
	Not in marital relationship	0.59 (0.33, 1.06)	0.078	0.59 (0.33, 1.06)	0.078

*Gamma_2 value is significant (p < 0.001) for the variable ‘Geographic location’; it violates the parallel-lines assumption of the POM

As shown in Table 3, the variable ‘geographic location’ violates the parallel-lines assumption of the proportional odds model. Therefore, the interpretation of the pattern of association of this variable with the outcome variable should be based on its unconstrained effect. Accordingly, highlanders compared to lowlanders showed a 0.23 (OR = 0.23; 95%CI: 0.10–0.57) times lower likelihood of having a high vs the combined medium and low dietary diversity. However, the likelihood of the combined high and moderate dietary diversity vs low dietary diversity was 1.17 (OR = 1.17; 95%CI: 0.74–1.84) times higher for highlanders as compared to lowlanders. Additionally, for a unit increase in food crops variety in the highlanders, that is, going from monocrop/no crops production to variety crops production and going from lowland to highland, the odds of having better dietary diversity was found to be 2.92 (OR = 2.92: 95%CI: 1.75–4.85) times higher given that the other variables fitted in the model are held constant.

## Discussion and conclusion

This study examined the burden and drivers of poor child nutrition among children aged 6–23 months in KA-HDSS, northern Ethiopia. The prevalence of child undernutrition (13.7 %) and inadequate child dietary diversity (< 4 food groups/day) (81.3%) was found to be high in our study setting. Key nutrition specific drivers of undernutrition included maternal undernutrition, inadequate access to child dietary diversity, and occurrence of morbidity. Furthermore, the principal nutrition-sensitive drivers of undernutrition were household poverty, larger family size, daily laborer occupation of household head, residing in highlands (≥ 2,300 meter) particularly where no diverse food crop production farming was practiced. Moreover, higher child dietary diversity (≥ 6 food groups/day) was positively associated with better wealth status and diverse food crops production.

In the study area, the prevalence of acute child undernutrition (MUAC<-2 z-score) was higher than the regional and national prevalence [9]. It is also considerably higher than the WHO’s 2025 target of ‘reduce and maintain childhood wasting to less than 5%’ [70]. Although the WHO’s target for wasting, which is based on WHZ<-2 z-score, is not identical with MUAC<-2 z-score, these two nutritional indices were shown to have similar performance in diagnosing acute child undernutrition [71]. Wasting among young children is associated with later detrimental effect on skeletal growth subsequent to linear growth retardation [72,73], and especially its concurrence with stunting, predicts high risk of child mortality [74,75]. Moreover, evidence on developmental origins of health and disease (DOHaD) revealed that survivors of severe acute undernutrition were reported to show traits of thrifty growth in later life [37], specifically, the association of early childhood infections and growth failure with elevated cardiovascular risk factors during adulthood is novel [34,36]. Considering the short and long-term deleterious impacts of wasting, addressing its key drivers could enormously enhance young child survival and wellbeing in the study setting.

The sex-specific analysis of undernutrition showed that boys were evidently more disadvantaged than girls. Our result accords with the findings of other studies [75–77], but it is inconsistent with the study from elsewhere [78], which also reported child age as an important factor. On the other hand, other studies from north Ethiopia reported no sex-differential of wasting, but of stunting [26,72]. Researchers explained that sex-specific differential burden of child undernutrition could be a manifestation of the complex interplay of the biologically higher vulnerability of males to symptomatic infectious illnesses and various context-specific factors such as cultural practices [26,79]. The apparently higher morbidity exposure of boys and relatively higher intake of quality diets by girls, reported in this study, might partly explain the observed nutritional inequality. Yet, further understanding of the underlying factors of the observed sex-specific inequality of child undernutrition, using qualitative research approach and longitudinal quantitative measurements accounting for the transitory nature of wasting, could have vital implications to public health nutrition policy and decisions aimed at addressing health inequity and ensuring nutritional wellbeing of all young children.

Adequate dietary diversity is a useful indicator of dietary quality and nutrient intake for infants and young children [18,51,80]. More than four-fifths of the participants of this study had no access to adequate dietary diversity, and this finding is markedly lower than the national estimation by Aemro et.al, which was 89.2% [19]. Similar to previous studies, inadequate access to child dietary diversity is found to be a strong predictor of child undernutrition [18,22]. Our finding shows a significantly lower consumption of some nutrient dense foods such as dairy, fruits and vegetables rich in vitamin A by the undernourished children. This might have co-exposed them to various deleterious risk factors (mainly infections like diarrheal diseases) that could influence their nutritional wellbeing [11,81]. In general, the study area is one of the most food insecure parts of Ethiopia, and pro-poor-oriented policy level developmental programs have been shown to be promising in ensuring household food security [82–84]. On top of such policy efforts, the implementation of home-based nutrition-sensitive interventions (home gardening, effective safety net programs together with asset and income-generating activities to poor households) and advancing maternal knowledge on child feeding practices may sustainably improve child nutritional status in such food insecure settings [5,12,38,81,83–87].

Many studies revealed that maternal nutritional status determines birth outcomes and neonatal survival, and has a pivotal role in fetal and child nutrition and development [2,12,29,81,88]. Consistent with these findings, our study indicates that children of undernourished mothers (MUAC < 23cm) were also more likely to suffer from undernutrition in contrast to those whose mothers were not undernourished. This may highlight the constitutive importance of promoting maternal-child dyad nutrition to substantially address young child undernutrition. The implementation of high impact maternal nutritional interventions could have indispensable inputs to reduce the burden of young child undernutrition and mortality [2,12,34]. Another notable finding of this study is the significant negative association of households’ morbidity experience with the young children’s nutritional wellbeing. Likewise, other studies identified that morbidity is considered to be one of the vital nutrition-specific determinant of child undernutrition, mainly due to its effect on dietary intake and nutrient utilization [11,25,27]. However, because of the limitation of our study design, we are not able to confirm if morbidity had resulted in child undernutrition or vice versa.

In line with other research findings [13,16,18,29,89], the current study has identified that higher household wealth status was associated with lower risk of child undernutrition. This association is independent of the effect of access to quality diet [18,90], a robust indicator of child undernutrition, and that wealth status may have influenced child nutritional status through multiple other pathways. As presented in our results, one such pathway could be the influence of wealth status on housing quality. This underscores the cross-functional role of household wealth on promoting young child nutrition by addressing the various nutrition-sensitive determinants of child undernutrition.

Higher family size was one of the strong nutrition-sensitive drivers of child undernutrition. This broadly substantiates the findings of previous studies [13,27,90,91]. In this study, the mean family size was differentially higher in the households where family planning (FP) was not utilized compared to the households where FP use was practiced (6.4 vs 5.9, p < 0.001). These findings imperatively call for making FP into effect to promote good nutritional status of mothers and their children [21,91]. The inverse association of household heads’ employment insecurity with undernutrition and poor child dietary diversity is direct [17,22]. As similarly reported by other studies [24,28], the burden of undernutrition was significantly higher among highlanders. Our data suggest better access to housing quality, media, sanitation, and electricity services by the low and/or midland households as compared to the highland households could have contributed to the higher burden of undernutrition among the highlanders as compared to their counterparts. However, the association of residing in highlands with child undernutrition strongly decreases in households which practiced diversified food crop production. This clearly shows that interventions targeting the agricultural sector to become nutrition-sensitive could potentially accelerate long-lasting effect for ameliorating child undernutrition, particularly if it is linked with the nutrition-specific interventions [12,38,92]. Despite our expectation, we did not find a statistically meaningful difference in the burden of child undernutrition by history of adult death, and the small adult death events in the study households might have limited the ability to detect its effect on child undernutrition.

Our study identified that inadequate child dietary diversity, maternal undernutrition and occurrence of morbidity were the robust nutrition-specific drivers, whereas, household poverty, household heads’ employment insecurity, larger family size, residing in highland households especially where no diverse food crops production was practiced were the strong nutrition-sensitive drivers of child undernutrition. The findings strongly suggest that intervention efforts aiming to reduce young child undernutrition should jointly address the examined nutrition-specific and sensitive drivers. For the success of such efforts in resource poor and food insecure rural settings, strong trans-sectoral collaborations and cross-disciplinary interventions, for instance the implementation of holistic developmental intervention programs and further interdisciplinary researches by the agriculture, nutrition and health sectors, could be highly important. The analysis of the effect of a wide range of possible drivers and the completion of the study before the 2016/17 el Nino shock, which may reflect the drivers of young child undernutrition under relatively stable conditions, are the major strengths of this study. However, due to the cross-sectional design of the study, the revealed relationships are only associational, and hence causality cannot be claimed.

## Paper context

Acute child undernutrition remains a persistently prevalent health problem in Tigray region, where over the course of 16 years, its burden showed no change. The prominent added value of this paper is that it has identified the nutrition-specific and sensitive determinants of young child undernutrition by examining a wide range of socio-economic, epi-demographic, agricultural, and other nutritional factors. Joint interventions by the agriculture, nutrition and health sectors are urgently needed to address young child undernutrition.

## References

[1] Troeger C, Colombara DV, Rao PC, et al Global disability-adjusted life-year estimates of long-term health burden and undernutrition attributable to diarrhoeal diseases in children younger than 5 years. Lancet Glob Health. 2018;6:e255-e269. 10.1016/S2214-109X(18)30045-7 PMC5861379 29433665

[2] Black RE, Victora CG, Walker SP, et al Maternal and child undernutrition and overweight in low-income and middle-income countries. Lancet. 2013;382:427–12. 23746772 10.1016/S0140-6736(13)60937-X

[3] Mulugeta A, Hagos F, Kruseman G, et al Child malnutrition in Tigray, northern Ethiopia. East Afr Med J. 2010;87:248–254. 23057267 10.4314/eamj.v87i6.63083

[4] Degarege D, Degarege A, Animut A. Undernutrition and associated risk factors among school age children in Addis Ababa, Ethiopia. BMC Public Health [Internet]. 2015;15:375. 10.1186/s12889-015-1714-5 PMC4411785 25879705

[5] Tessema M, Belachew T, Ersino G Feeding patterns and stunting during early childhood in rural communities of Sidama, South Ethiopia. Pan Afr Med J. 2013;14:75. 23646211 10.11604/pamj.2013.14.75.1630 PMC3641921

[6] Fikadu T, Assegid S, Dube L Factors associated with stunting among children of age 24 to 59 months in Meskan district, Gurage Zone, South Ethiopia: A case-control study. BMC Public Health. 2014;14. 10.1186/1471-2458-14-800 PMC4131046 25098836

[7] Woodruff BA, Wirth JP, Bailes A, et al Determinants of stunting reduction in Ethiopia 2000 – 2011. Matern Child Nutr. 2017;13. 10.1111/mcn.12307 PMC6866086 27161654

[8] Haile D, Azage M, Mola T, et al Exploring spatial variations and factors associated with childhood stunting in Ethiopia: spatial and multilevel analysis. BMC Pediatr. 2016;16. 10.1186/s12887-016-0587-9 PMC4833938 27084512

[9] Central Statistical Agency [Ethiopia] and ICF International Ethiopia demographic and health survey 2016 key indicators report. Addis Ababa, Ethiopia, and Rockville. Maryland: USA. CSA and ICF; 2016.

[10] Central Statistical Authority [Ethiopia] and ORC Macro Ethiopia demographic and health survey 2000. Addis Ababa: Central Statistical Authority and ORC Macro; 2000.

[11] UNICEF Strategy for improved nutrition of children and women in developing countries. Policy Rev. Pap. E/ICEF/1990/1.6. New York: UNICEF; 1990.

[12] Bhutta ZA, Das JK, Rizvi A, et al Maternal and child nutrition 2: evidence-based interventions for improvement of maternal and child nutrition : what can be done and at what cost? Lancet. 2013;6736:1–26. 10.1016/S0140-6736(13)60996-4 23746776

[13] Victora CG, Vaughan JP, Kirkwood BR, et al Risk factors for malnutrition in Brazilian children: the role of social and environmental variables. Bull World Health Organ. 1986;64:299–309. 3488846 PMC2490948

[14] Ali D, Saha KK, Nguyen PH, et al Household food insecurity is associated with higher child undernutrition in Bangladesh, Ethiopia, and Vietnam, but the effect is not mediated by child dietary diversity. J Nutr. 2013;143:2015–2021. 24089419 10.3945/jn.113.175182

[15] Smith LC, Haddad L Reducing child undernutrition: past drivers and priorities for the post-MDG era. World Dev. 2015;68:180–204.

[16] Petrou S, Kupek E Poverty and childhood undernutrition in developing countries: A multi-national cohort study. Soc Sci Med. 2010;71:1366–1373. 20691525 10.1016/j.socscimed.2010.06.038

[17] Negash C, Whiting SJ, Henry CJ, et al Association between maternal and child nutritional status in Hula, rural Southern Ethiopia: A cross sectional study. PLoS One. 2015;10. 10.1371/journal.pone.0142301 PMC4654505 26588687

[18] Arimond M, Ruel MT Dietary diversity is associated with child nutritional status: evidence from 11 demographic and health surveys. J Nutr. 2004;134:2579–2585. 15465751 10.1093/jn/134.10.2579

[19] Aemro M, Mesele M, Birhanu Z, et al Dietary diversity and meal frequency practices among infant and young children aged 6 – 23 months in Ethiopia: a secondary analysis of ethiopian demographic and health survey 2011. J Nutr Metab. 2013;2013:8. 10.1155/2013/782931 PMC3878383 24455218

[20] Pongou R, Ezzati M, Salomon JA Household and community socioeconomic and environmental determinants of child nutritional status in Cameroon. BMC Public Health. 2006;6. 10.1186/1471-2458-6-98 PMC1523206 16618370

[21] Ighogboja SI Some factors contributing to protein-energy malnutrition in the middle belt of Nigeria. East Afr Med J. 1992;69:566–571. 1473511

[22] Tariku A, Bikis GA, Woldie H, et al Child wasting is a severe public health problem in the predominantly rural population of Ethiopia: A community based cross-sectional study. Arch Public Health. 2017;75. 10.1186/s13690-017-0194-8 PMC5467055 28616226

[23] Kumar N, Harris J, Rawat R If they grow it, will they eat and grow? Evidence from zambia on agricultural diversity and child undernutrition. J Dev Stud. 2015;51:1060–1077.

[24] Dang S, Yan H, Yamamoto S, et al Poor nutritional status of younger Tibetan children living at high altitudes. Eur J Clin Nutr. 2004;58:938–946. 15164115 10.1038/sj.ejcn.1601915

[25] Wamani H, Nordrehaug AS, Peterson S, et al Predictors of poor anthropometric status among children under two years of age in rural Uganda. Public Health Nutr. 2006;9:320–326. 16684383 10.1079/phn2006854

[26] Abebe Z, Zelalem Anlay D, Biadgo B, et al High prevalence of undernutrition among children in Gondar Town, Northwest Ethiopia: a community-based cross-sectional study. Int J Pediatr. 2017;2017:1–9. 10.1155/2017/5367070 PMC5745770 29387093

[27] Basit A, Nair S, Chakraborthy KB, et al Risk factors for under-nutrition among children aged one to five years in Udupi taluk of Karnataka, India: a case control study. Australas Med J. 2012;5:163–167. 22952561 10.4066/AMJ.20121022 PMC3433731

[28] Katuli S, Natto ZS, Beeson L, et al Nutritional status of highland and lowland children in ecuador. J Trop Pediatr. 2013;59:3–9. 22752465 10.1093/tropej/fms032

[29] Assefa N, Berhane Y, Worku A Wealth status, mid upper arm circumference (MUAC) and Ante Natal Care (ANC) are determinants for low birth weight in Kersa, Ethiopia. PLoS One. 2012;7:e39957. 10.1371/journal.pone.0039957 PMC3386987 22792140

[30] Pryer J The impact of adult ill-health on household income and nutrition in Khulna, Bangladesh. Environ Urban. 1993;5:35–49.

[31] Mirelman AJ, Rose S, Khan JA, et al The relationship between non-communicable disease occurrence and poverty - evidence from demographic surveillance in Matlab, Bangladesh. Health Policy Plan. 2016;31:785–792. 26843515 10.1093/heapol/czv134

[32] Khan JAM, Trujillo AJ, Ahmed S, et al Distribution of chronic disease mortality and deterioration in household socioeconomic status in rural Bangladesh: an analysis over a 24-year period. Int J Epidemiol. 2015;44:1917–1926. 26467760 10.1093/ije/dyv197 PMC5156339

[33] Perez-Escamilla R, Dessalines M, Finnigan M, et al Household food insecurity is associated with childhood malaria in rural Haiti. J Nutr. 2009;139:2132–2138. 19741201 10.3945/jn.109.108852

[34] Victora CG, Adair L, Fall C, et al Maternal and child undernutrition: consequences for adult health and human capital. Lancet. 2008;371:340–357. 18206223 10.1016/S0140-6736(07)61692-4 PMC2258311

[35] Grantham-McGregor S, Cheung YB, Cueto S, et al Developmental potential in the first 5 years for children in developing countries. Lancet. 2007;369:60–70. 17208643 10.1016/S0140-6736(07)60032-4 PMC2270351

[36] Deboer MD, Lima AAM, Oría RB, et al Early childhood growth failure and the developmental origins of adult disease: do enteric infections and malnutrition increase risk for the metabolic syndrome? Nutr Rev. 2012;70:642–653. 23110643 10.1111/j.1753-4887.2012.00543.x PMC3493112

[37] Lelijveld N, Seal A, Wells JC, et al Chronic disease outcomes after severe acute malnutrition in Malawian children (ChroSAM): a cohort study. Lancet Glob Heal. 2016;4:e654–e662. 10.1016/S2214-109X(16)30133-4 PMC4985564 27470174

[38] Ruel MT, Alderman H Nutrition-sensitive interventions and programmes: how can they help to accelerate progress in improving maternal and child nutrition? Lancet. 2013;382:536–551. 23746780 10.1016/S0140-6736(13)60843-0

[39] Victora CG, de Onis M, Hallal PC, et al Worldwide timing of growth faltering: revisiting implications for interventions. Pediatrics. 2010;125:e473–e480. 20156903 10.1542/peds.2009-1519

[40] Abera SF, Gebru AA, Biesalski HK, et al Social determinants of adult mortality from non-communicable diseases in northern Ethiopia, 2009-2015: evidence from health and demographic surveillance site. PLoS One. 2017;12. 10.1371/journal.pone.0188968 PMC5728486 29236741

[41] Weldearegawi B, Ashebir Y, Gebeye E, et al Emerging chronic non-communicable diseases in rural communities of Northern Ethiopia: evidence using population-based verbal autopsy method in Kilite Awlaelo surveillance site. Health Policy Plan. 2013;28:891–898. 23293101 10.1093/heapol/czs135

[42] Praveen K, Neha S, Sutapa A, et al Screening maternal acute malnutrition using adult mid-upper arm circumference in resource-poor settings. Indian J Community Med. 2018;43:132–134. 29899619 10.4103/ijcm.IJCM_248_17 PMC5974833

[43] Bisai S, Bose K Undernutrition in the Kora Mudi tribal population, West Bengal, India: A comparison of body mass index and mid-upper-arm circumference. Food Nutr Bull. 2009;30:63–67. 19445260 10.1177/156482650903000106

[44] Tang AM, Dong K, Deitchler M, et al Use of cutoffs for Mid-Upper Arm Circumference (MUAC) as an indicator or predictor of nutritional and health related outcomes in adolescents and adults: a systematic review. Washington, DC: FHI 360/FANTA; 2013.

[45] De Onis M, Yip R, Mei Z The development of MUAC-for-age reference data recommended by a WHO expert committee. Bull World Health Organ. 1997;75:11–18. 9141745 PMC2486977

[46] WHO Physical status: the use and interpretation of anthropometry. report of a WHO expert committee. 1995 p. 1–452. (Technical Report Series No. 854. Phys. status use Interpret. Anthr. Rep. a WHO Expert Committee. Tech. Rep. Ser. No. 854). 8594834

[47] De Onis M, Habicht JP Anthropometric reference data for international use: recommendations from a World Health Organization Expert Committee. Am J Clin Nutr. 1996;64:650–658. 8839517 10.1093/ajcn/64.4.650

[48] Hall G, Chowdhury S, Bloem M Use of mid-upper-arm circumference Z scores in nutritional assessment. Lancet. 1993;341:1481. 10.1016/0140-6736(93)90927-9 8099180

[49] Custodio E, Martin-Cañavate R, Marcantonio FD, et al MUAC-for-age more useful than absolute MUAC for nutritional surveillance in Somalia: results from nineteen cross-sectional surveys (2007–2016). BMC Nutr. 2018;4. 10.1186/s40795-018-0213-3 PMC7050741 32153872

[50] World Health Organization WHO Anthro for personal computers, version 3.2.2: software for assessing growth and development of the world’s children. Geneva: World Health Organization; 2011 p. 1–57.

[51] Dewey KG, Cohen RJ, Arimond M, et al Developing and validating simple indicators of complementary food intake and nutrient density for breastfed children in developing countries. Maturitas. 2007;58:150–155.17768019

[52] World Health Organization Indicators for assessing infant and young child feeding practices-Part 1 definitions. Washington, DC: World Health Organization; 2007.

[53] FAO Guidelines for measuring household and individual dietary diversity. Rome, Italy: FAO; 2010.

[54] Kuku-Shittu O, Onabanjo O, Fadare O, et al Child malnutrition in Nigeria: evidence from Kwara State. Int Food Policy Res Inst. 2016;1–64.

[55] Ochieng J, Afari-Sefa V, Lukumay PJ, et al Determinants of dietary diversity and the potential role of men in improving household nutrition in Tanzania. PLoS One. 2017;12:e0189022. 10.1371/journal.pone.0189022 PMC5726653 29232413

[56] Howe LD, Galobardes B, Matijasevich A, et al Measuring socio-economic position for epidemiological studies in low-and middle-income countries: a methods of measurement in epidemiology paper. Int J Epidemiol. 2012;41:871–886. 22438428 10.1093/ije/dys037 PMC3396323

[57] McKenzie DJ Measuring inequality with asset indicators. J Popul Econ. 2005;18:229–260.

[58] World Health Organization/UNICEF Progress on drinking water and sanitation: 2015 update. Geneva: World Health Organization; 2015.

[59] Adebowale SA, Morakinyo OM, Ana GR Housing materials as predictors of under-five mortality in Nigeria: evidence from 2013 demographic and health survey. BMC Pediatr. 2017;17:30. 10.1186/s12887-016-0742-3 PMC5248529 28103828

[60] World Health Organization. International Statistical Classification of Diseases and Related Health Problems 10th Revision (ICD-10). Geneva: World Health Organization; 2010.

[61] Golics CJ, Basra MKA, Salek MS, et al The impact of patients’ chronic disease on family quality of life: an experience from 26 specialties. Int J Gen Med. 2013;6:787–798. 24092994 10.2147/IJGM.S45156 PMC3787893

[62] Van Minh H, Xuan Tran B Assessing the household financial burden associated with the chronic non-communicable diseases in a rural district of Vietnam. Glob Health Action. 2012;5:1–7. 10.3402/gha.v5i0.18892 PMC3529202 23273250

[63] Zhang J, Yu KF What’s the relative risk? A method of correcting the odds ratio in cohort studies of common outcomes. JAMA. 1998;280:1690–1691. 9832001 10.1001/jama.280.19.1690

[64] Cummings P Methods for estimating adjusted risk ratios. Stata J. 2009;9:175–196.

[65] Das S, Rahman RM Application of ordinal logistic regression analysis in determining risk factors of child malnutrition in Bangladesh. Nutr J. 2011;10. 10.1186/1475-2891-10-124 PMC3296641 22082256

[66] Williams R Understanding and interpreting generalized ordered logit models. J Math Sociol. 2016;50:7–20.

[67] O’Brien RM A caution regarding rules of thumb for variance inflation factors. Qual Quant. 2007;41:673–690.

[68] Mickey RM, Greenland S The impact of confounder selection criteria on effect estimation. Am J Epidemiol. 1989;129:125–137. 2910056 10.1093/oxfordjournals.aje.a115101

[69] Zhang Z Model building strategy for logistic regression: purposeful selection. Ann Transl Med. 2016;4:111. 27127764 10.21037/atm.2016.02.15 PMC4828741

[70] World Health Organization Global nutrition targets 2025 policy brief series. Geneva: World Health Organization; 2014.

[71] Mwangome MK, Fegan G, Fulford T, et al Mid-upper arm circumference at age of routine infant vaccination to identify infants at elevated risk of death: a retrospective cohort study in the Gambia. Bull World Health Organ. 2012;90:887–894. 23284194 10.2471/BLT.12.109009 PMC3524961

[72] Kiyu A, Teo B, Hardin S, et al Nutritional status of children in rural Sarawak, Malaysia. Southeast Asian J Trop Med Public Health. 1991;22:211–215. 1948281

[73] Saaka M, Galaa SZ Relationships between wasting and stunting and their concurrent occurrence in Ghanaian preschool children. J Nutr Metab. 2016;2016:1–11. 10.1155/2016/4654920 PMC4917721 27379184

[74] O’Neill M, Fitzgerald S, Briend A, et al Child mortality as predicted by nutritional status and recent weight velocity in children under two in rural Africa. J Nutr. 2012;142:520–525. 22259194 10.3945/jn.111.151878

[75] Khara T, Mwangome M, Ngari M, et al. Children concurrently wasted and stunted: a meta-analysis of prevalence data of children 6-59 months from 84 countries. Matern Child Nutr. 2018;14:e12516. 10.1111/mcn.12516 PMC5901398 28944990

[76] Wamani H, Åstrøm AN, Peterson S, et al Boys are more stunted than girls in Sub-Saharan Africa: A meta-analysis of 16 demographic and health surveys. BMC Pediatr. 2007;7. 10.1186/1471-2431-7-17 PMC1865375 17425787

[77] Badake QD, Maina I, Mboganie MA, et al Nutritional status of children under five years and associated factors in Mbeere South District, Kenya. African Crop Sci J. 2014;22:799–806.

[78] Olack B, Burke H, Cosmas L, et al Nutritional status of under-five children living in an informal urban settlement in Nairobi, Kenya. J Heal Popul Nutr. 2011;29:357–363. 10.3329/jhpn.v29i4.8451 PMC3190366 21957674

[79] Green N The male predominance in the incidence of infectious disease in children: A postulated explanation for disparities in the literature. Int J Epidemiol. 1992;21:381–386. 1428496 10.1093/ije/21.2.381

[80] Moursi MM, Arimond M, Dewey KG, et al Dietary diversity is a good predictor of the micronutrient density of the diet of 6- to 23-month-old children in Madagascar. J Nutr. 2008;138:2448–2453. 19022971 10.3945/jn.108.093971

[81] Bhutta ZA, Ahmed T, Black RE, et al Maternal and child undernutrition 3 what works? Interventions for maternal and child undernutrition and survival. Lancet. 2008;371:417–440. 18206226 10.1016/S0140-6736(07)61693-6

[82] van der Veen A, Gebrehiwot T Effect of policy interventions on food security in Tigray, Northern Ethiopial. Ecol Soc. 2011; 16:18.

[83] Hoddinott J, Berhane G, Gilligan DO, et al The impact of Ethiopia’s productive safety net programme and related transfers on agricultural productivity. J Afr Econ. 2012;21:761–786.

[84] Gilligan DO, Hoddinott J, Kumar N, et al Impact of social protection on food security and coping mechanisms: evidence from Ethiopia’s productive safety nets program. J Vestib Res. 2011;21:297–298.22348933

[85] Cabalda AB, Rayco-Solon P, Solon JAA, et al Home gardening is associated with Filipino preschool children’s dietary diversity. J Am Diet Assoc. 2011;111:711–715. 21515117 10.1016/j.jada.2011.02.005

[86] Bhutta ZA Micronutrient needs of malnourished children. Curr Opin Clin Nutr Metab Care. 2008;11:309–314. 18403929 10.1097/MCO.0b013e3282fbf5a0

[87] Olney DK, Talukder A, Iannotti LL, et al Assessing impact and impact pathways of a homestead food production program on household and child nutrition in Cambodia. Food Nutr Bull. 2009;30:355–369. 20496626 10.1177/156482650903000407

[88] Kramer MS, Kakuma R Energy and protein intake in pregnancy. Cochrane Database Syst Rev. 2003;CD000032. 14583907 10.1002/14651858.CD000032

[89] Rahman MM, Saima U, Goni MA Impact of maternal household decision-making autonomy on child nutritional status in Bangladesh. Asia-Pacific J Public Health. 2015;27:509–520. 10.1177/1010539514568710 25657298

[90] Headey DD Developmental drivers of nutritional change: a cross-country analysis. World Dev. 2013;42:76–88.

[91] Conde-Agudelo A, Rosas-Bermudez A, Castano F, et al Effects of birth spacing on maternal, perinatal, infant, and child health: a systematic review of causal mechanisms. Stud Fam Plann. 2012;43:93–114. 23175949 10.1111/j.1728-4465.2012.00308.x

[92] Swaminathan MS Zero hunger. Science. 2014;345:491. 25082671 10.1126/science.1258820

