# Supplementary material for: Nutrition-specific and sensitive drivers of poor child nutrition in Kilte Awlaelo-Health and Demographic Surveillance Site, Tigray, Northern Ethiopia: implications for public health nutrition in resource-poor settings
Source: Glob Health Action. 2019 Jan 14;12(1):1556572. doi: 10.1080/16549716.2018.1556572 (PMC6338276; doi:10.1080/16549716.2018.1556572)
Supplement: Supplemental Material [file ZGHA_A_1556572_SM4956.rtf]

                                          


Supplementary Materials


Appendix A: Descriptive summary of wealth status by household characteristics 
Table A1. Distribution of wealth index values by housing characteristics, ownership of durable assets and access to different services, KA-HDSS, Tigray, northern Ethiopia (n=1, 483)
Characteristics	Categories	Row distribution of wealth index in percent  	Total, n (%)	
		Quintile 1	Quintile 2	Quintile 3	Quintile 4	Quintile 5		
Poor livestock and crops ownership	Yes	20.6	16.7	17.2	17.5	28.0	593 (40.0)	
	No	20.0	22.2	21.7	22.4	13.7	890 (60.0)	
Farmland 	No	5.1	4.5	15.3	20.7	54.4	333 (22.5)	
	Yes 	24.6	24.5	21.2	20.4	9.3	1, 150 (77.5)	
Farmland size	≤2 ha	19.1	17.2	19.8	20.5	23.4	1, 139 (76.8)	
	>2ha	24.1	29.3	20.1	20.1	6.4	344 (21.2)	
Floor	Unimproved	22.0	21.8	21.6	22.0	12.6	1, 365 (92.0)	
	Improved	0.0	0.0	0.0	1.7	98.3	118 (8.0)	
Wall	Unimproved	25.0	24.0	21.1	16.8	13.1	1, 202 (81.1)      	
	Improved	0.0	2.9	14.6	35.9	46.6	281 (18.9)	
Roof  	Unimproved	17.3	21.9	24.4	24.2	12.2	842 (56.8)	
	Improved	24.0	17.6	14.0	15.5	28.9	641 (43.2)	
Access to electricity 	No	23.6	23.2	21.7	21.0	10.4	1, 265 (85.3)	
	Yes	0.5	1.4	9.2	17.4	71.5	218 (14.7)	
Access to Media  	No	12.2	20.1	24.6	22.3	20.8	452 (30.5)	
	Yes	23.8	20.0	17.8	19.6	18.8	1, 031 (69.5)	
Kitchen	No	45.9	14.9	9.3	9.3	20.7	410 (27.6)	
	Yes	10.4	22.0	24.0	24.7	18.9	1, 073 (72.4)	
Access to
improved water 	No	27.3	20.4	20.9 	24.4	7.0	201 (13.6)	
	Yes	19.1	20.0	19.7	19.8	21.4	1, 282 (86.4)	
Water fetching duration 	≥30 minutes	11.9	22.1	24.6	26.2	15.2	711 (47.9)	
	<30 minutes	27.9	18.1	15.5	15.2	23.2	772 (52.1)	
Access to improved 
latrine	No	21.4	17.5	17.0	23.8	20.3	1, 142 (77.0)	
	Yes	16.4	28.5	29.6	9.1	16.4	341 (23.0)	
Owned bed with sofa	No	18.2	20.5	24.4	23.8	13.1	908 (61.2)	
	Yes	23.5	19.3	12.7	15.1	29.4	575 (38.8)	
Own bee hive 	No	19.6	19.1	19.3	20.4	21.6	1, 258 (84.8)	
	Yes	24.0	25.3	23.1	20.4	7.1	225 (15.2)	
Cooking fuel  	biomass	20.9	20.7	20.6	21.1	16.7	1, 434 (96.7)	
	non-biomass	0.0	0.0	0.0	0.0	100.0	49 (3.3)	
Sleeping room attached to neighbor's house	Yes	0.0	1.6	7.4	33.1	57.9	435 (29.3)	
	No	28.6	27.7	25.1	15.2	3.4	1, 048 (70.7)	


Appendix B: Child MUAC Z score distribution 

Figure B1.   Distribution of Z score-based child MUAC, eastern Tigray, northern Ethiopia (n=1, 525)


Appendix C: Summary of child dietary diversity by selected characteristics 
Table C1. Dietary diversity of children aged 6 to 23 months by selected independent variables, KA-HDSS, Tigray, northern Ethiopia (n=1, 525)             
Characteristics	Categories	Row distribution of Child dietary 
diversity (7 food groups/day) in %	Total n (%)	
		Low
(≤3 food groups)	Medium
(4-5 food groups)	High
(≥6 food groups)		
Sex of child	Female	81.0 	15.6	3.4	770 (50.5)	
	Male	81.4	15.0	3.6	755 (49.5)	
Residence	Semi-urban	84.1	15.3	0.0	85 (5.6)	
	Rural	81.0	15.3	3.7	1, 440 (94.4)	
Marital status of household head	Married	80.6	15.7	3.7	1, 387 (90.9)	
	Single/divorced/widowed	87.6	11.0	1.4	137 (9.0)	
	Missing				1 (0.01)	
Occupation of household head 	Farmer/housewife	80.6	15.5	3.9	1, 292 (84.7)	
	Daily laborer	85.3	13.3	1.4	143 (9.4)	
	Government employee and all others	83.3	15.6	1.1	90 (5.9)	
History of adult death 	No history adult death	81.4	15.0	3.6	1, 434 (94.0)	
	Death from chronic diseases	77.8	20.0	2.2	45 (3.0)	
	Death from acute infectious diseases, injuries and other causes	80.4	17.4	2.2	46 (3.0)	
Wealth status	Poor	87.8	9.4	2.8	597 (39.1)	
	Medium	83.7	13.9	2.4	295 (19.3)	
	Wealthy	73.6	21.8	4.6	591 (38.8)	
	Missing				42 (2.8)	
Geographic location	Low/midland	84.4	11.5	4.1	1, 038 (68.1)	
	Highland	70.4	27.5	2.1	375 (24.6)	
	missing				112 (7.3)	
Maternal health seeking practice	Poor practice	81.7	14.7	3.6	1, 153 (75.6)	
	Good practice	79.5	17.4	3.1	361 (23.7)	
	Missing				11 (0.7)	


Appendix D: Methodology
Wealth index variable was generated from accessibility to improved water and sanitation services as defined by the WHO/UNICEF Joint Monitoring Program (JMP) ladders [58], housing quality levels, access to electricity, media (created from single or joint ownership of TV or radio or mobile phone or home phone), availability of kitchen, use of non-biomass energy source for food cooking, ownership of farmland, farmland size, bed with sofa, , duration to fetch water (good if the round duration is <30 minutes), bee hive, etc. In addition to these attributes, a quintile position (later dummied) based on agricultural crop production, livestock and other rarely or mostly owned assets, converted to Ethiopian birr (ETB) were added in the estimation of wealth index. The agricultural crop productions (maize, teff, wheat, sorghum, and legumes), ownership of livestock animals (like cow, ox, calf, sheep, goat, camel, donkey, horse, mule, and chicken) and other assets were converted to ETB based on the estimated local transaction prices of these assets during the survey time. The prices of the two assets (animals and food crops) were summed, adjusted for the size of household members and then quintile classified treating the lowest two categories as “agricultural asset poor” indicator. This dummy variable has helped us for addressing the main limitation of principal component analysis (PCA), i.e., this approach helped us to consider every mostly owned or rarely owned  asset variables to be included in this proxy indicator without the need of dropping such variables from the analysis due to their underlying inability to differentiate the households' status, a potential problem of PCA that might have been observed had these variables been included in the PCA procedure as separate indicators [57]. Due to this fact, the approach quantifies assets better and reflects the 'actual' asset status of each household.  
The classifications of the quality of floor, wall and roof of the houses (as “improved” or “unimproved” was derived from the multiple and exhaustive types of floor, wall and roof) were replicating a prior research work [59]. These variables were then factors analyzed using PCA, a multivariate data reduction procedure commonly used in measuring socio-economic position, and quintile classified considering the five principal components to generate this latent variable [56]. These five principal components were selected because the corresponding eigen values were above 1 as shown by the scree plot (Figure E1). Finally, wealth index was constructed for 1, 483 (97.2%) households, and it was missed for the remaining 42 (2.8%) households due to the fact there was at least one missing value in any of the factorized observable variable.

Appendix E: Principal components with eigen values greater than 1

Figure E1. Five selected principal components (with eigen value >1) used for wealth index construction


Appendix F: Multicollinearity test using variance inflation factor (VIF)

Figure F1. Diagnostic statistical test for multicollinearity as measured in variance inflation factor (VIF)
